# Nanotechnology in orthopedics: a clinically oriented review

**DOI:** 10.1186/s12891-018-1990-1

**Published:** 2018-03-02

**Authors:** Walter Ryan Smith, Parke William Hudson, Brent Andrew Ponce, Sakthivel Rajan Rajaram Manoharan

**Affiliations:** 1 Department of Orthopaedic Surgery, University of Alabama at Birmingham, 1313 13 St. South, Birmingham, AL 35205 USA; 2 Department of Orthopaedic Surgery, University of Alabama at Birmingham, FOT 950, 20 Street South, Birmingham, AL 35294 USA

## Abstract

The utility of nanotechnology in medicine, specifically within the field of orthopedics, is a topic of extensive research. Our review provides a unique comprehensive overview of the current and potential future uses of nanotechnology with respect to orthopedic sub-specialties. Nanotechnology offers an immense assortment of novel applications, most notably the use of nanomaterials as scaffolds to induce a more favorable interaction between orthopedic implants and native bone. Nanotechnology has the capability to revolutionize the diagnostics and treatment of orthopedic surgery, however the long-term health effects of nanomaterials are poorly understood and extensive research is needed regarding clinical safety.

## Background

Disruptive technology and innovation have long held the promise of improving patient outcomes. The field of nanotechnology is one of these domains with breakthrough potential to aid in diagnosing and treating complex medical problems. Nanotechnology was originally defined by the National Nanotechnology Initiative as the study and controlled manipulation of individual atoms and molecules of size between 1 and 100 nm, however the definition has since evolved to include a broader spectrum of research endeavors and applications [1]. Richard Feynman was the first to conceptualize the potential of nanotechnology nearly six decades ago. In 1959, he described it as “a field in which little has been done, but in which an enormous amount can be done in principle [2].” Since then, the applications of nanotechnology have vastly expanded into fields such as food packaging, cosmetics, water filtration, and medicine [3].

The application of nanotechnology to medicine, known as “nanomedicine,” has been utilized in a number of novel therapies in the field of orthopedics. A few clinical applications include targeted drug delivery, implantable materials, vertebral disk regeneration, and diagnostic modalities [4]. Previous reviews of nanotechnology in orthopedics have provided extensive summaries regarding the use of different biomaterials that have been studied and implemented [5, 6]. Our review is unique in the sense that it is structured by orthopedic sub-specialty with a specific focus on the clinical aspects of nanotechnology in orthopedics. While specialty designations are arbitrary, they are useful to highlight the clinical relevance of certain innovations with appreciation that cross over applications into other sub-specialties are likely in the future. Our main objective is to provide orthopedic surgeons and musculoskeletal researchers with knowledge regarding the current impact and future potential of nanotechnology. We will also identify nanotechnology studies with ongoing clinical trials in each respective section.

### Basics of nanotechnology

**Fig. 1:**
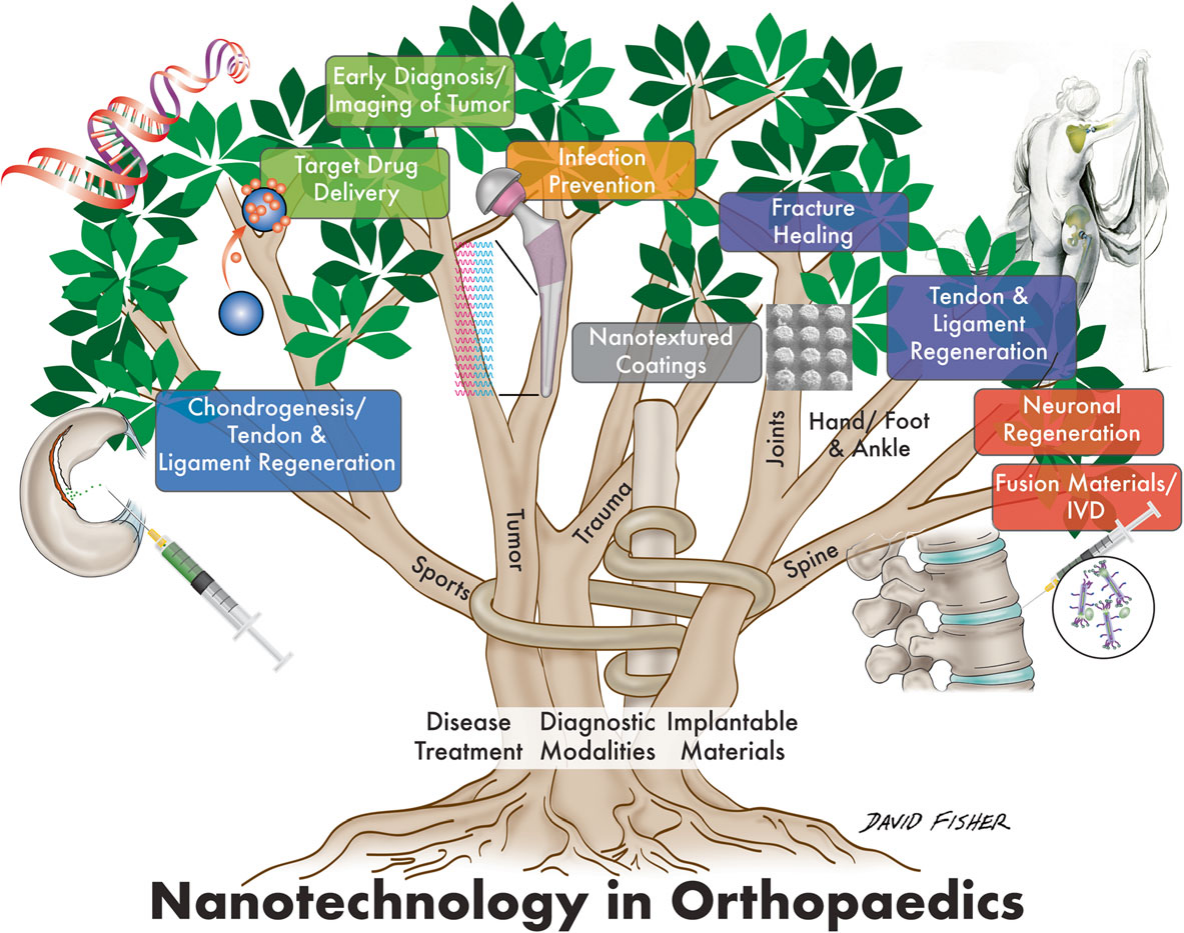
Diagram outlining the domains and applications of nanotechnology within orthopedics

Nanotechnology exists as a collaboration among multiple scientific disciplines including surface science, molecular biology, microelectronics, and tissue engineering. When conventional macro-materials are engineered into much smaller nanosized particles, they may possess completely different physical and chemical properties in certain instances. Specifically, as the size of particulate matter decreases to 100 nm or smaller, phenomena such as the quantum size effect become more prominent [7]. This principle is observed when the electrical properties of a material change as a result of significant reductions in particle size. For example, materials that are insulators at the macroscale may possess conductive properties when reduced to the nanoscale. In addition to alterations in electrical properties, changes in mechanical properties may take place as well due to an increased surface area to volume ratio as particle size is reduced. This bears significance as nanophase materials are able to maintain relatively large surface area to volume ratios allowing for more favorable interactions with surrounding structures. In the example of orthopedic implants, this allows for a greater degree of interaction between an implant and native bone, leading to more effective osseointegration [8]. Much of nanotechnologies’ potential benefit with regards to medicine rests in the fact that nanotechnology may allow for more precise therapeutic applications at the subcellular level [9]. Given that many molecules involved in cellular processes exist and interact fundamentally at the nanometer scale, nanoengineered materials have the theoretical ability to target and modify these processes [10]. Applying this principle to orthopedics, bone when broken down to the nanoscale is naturally a nanostructure composite of collagen and hydroxyapatite [11]. The practical application of these principles and appreciation of these relationships has allowed for improvements in functionality and performance of a wide variety of products both in and outside of the medical field. Figure 1 summarizes the potential utilities of nanotechnology among different orthopedic sub-specialties.

## Spine

### Tissue regeneration

Surgical treatments for degenerative disc disease such as discectomy and fusion are often associated with the loss of spinal mobility, degenerative post-discectomy spondylosis, and disc herniation recurrence [12]. Inconsistent outcomes and complications with current treatments have created a role for nanotech research involving novel cell-based therapies, including tissue engineering for intervertebral disc (IVD) regeneration. These experimental therapies have demonstrated the ability of progenitor cells, such as mesenchymal stem cells (MSCs) to undergo differentiation into a nucleus pulposus-like phenotype [13–16]. Injection therapy with poly (γ-glutamic acid) nanocomplexes has been shown in multiple studies to enhance recovery of native IVD matrix [13, 14]. Additionally, these nanocomplexes have demonstrated anti-inflammatory properties in ex vivo models. Growth factors are often used concurrently to promote proliferation and differentiation. However, one of the underlying problems with this technique is that short lives in vivo limit their utility. To address this issue, current studies are aiming to develop nanofibrous scaffolds to help sustain biologically active growth factors and maximize the potential of MSCs. The combination of these scaffolds with growth factors such as TGF-β under certain conditions has shown promising results in achieving a functional graft for IVD regeneration [15]. These advances in scaffold engineering, while still relatively new, may offer an efficient method for nucleus pulposus regeneration.

**Fig. 2:**
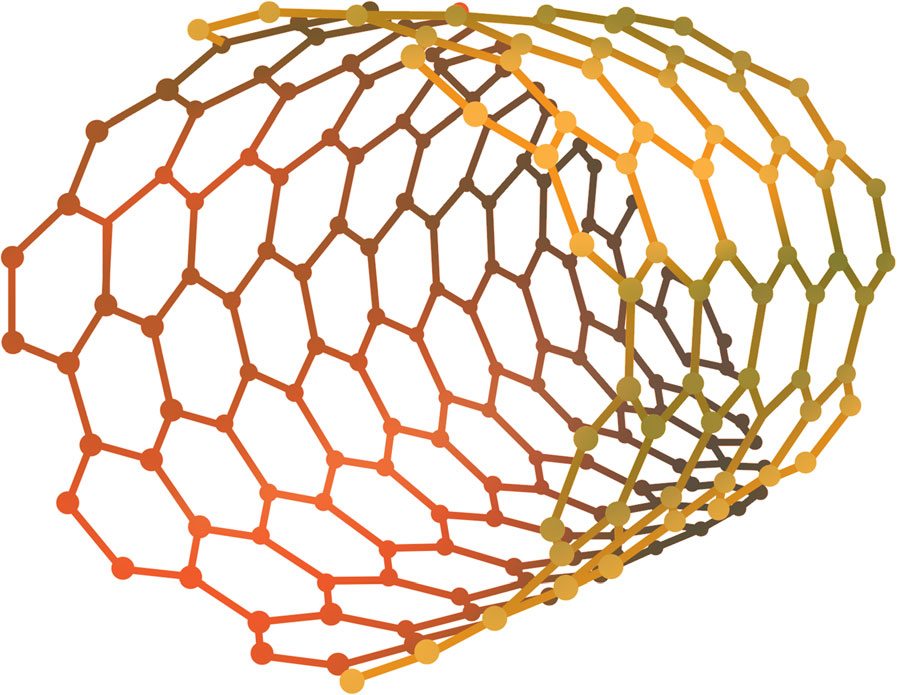
Illustration showing the basic structure of a carbon nanotube. These materials are of significant interest in multiple realms of orthopedics including nerve regeneration, implant scaffold engineering, and drug delivery

Surgical interventions for peripheral nerve injuries have been criticized in the past as inefficient and cost-defective. Neuron regeneration using nanoengineering may offer an attractive method for management of peripheral nerve injuries by eliminating the morbidity associated with surgical interventions to harvest an autograft. Synthetic conduits using carbon nanotubes and nanoscaffolds offer more tunable mechanical properties than autografts and may enhance nerve regeneration through augmented surface topographical interactions [17]. Carbon nanotubes have demonstrated the ability to promote axonal growth and even mimic some electrical properties of myelin (Fig. 2) [9]. Electrospinning techniques have been paramount in allowing for the creation of nanoscaffolds, which closely resemble native extracellular environments. [18] The nanoscale fibers that comprise these conduits have increased area available for protein absorption, stem cell migration, and axonal regeneration and guided growth in addition to being easily and cheaply manufactured [17]. Commonly used materials in conduit composition include collagen and silicone. Although not yet clinically mainstream, nanoscale synthetic nerve conduits appear to be the future of nerve regeneration therapy. Artificial conduits have the potential to replace autografting as first-line therapy and may even allow surgeons to tailor conduits according to the type of nerve, sensory, motor or mixed, needed.

### Spinal implants

In addition to disc and nerve regeneration, nanotechnology may potentially facilitate spinal fusion and avoid the cost and potential complications associated with recombinant human bone morphogenetic protein (rhBMP). Surface modifications to titanium spinal implants through the addition of nanoparticles such as titanium oxide and zirconia have shown promise in promoting increased bone formation and decreased resorption compared to conventional smooth implants. [19] Additionally, cervical cages enhanced with silicone nitride nanoparticles have demonstrated multiple biomechanical advantages over standard PEEK (poly-ether-ether-ketone) and are currently on the market [20]. In 2014, the FDA approved the first interbody fusion device to feature nanotechnology [21]. The nanoLOCK™ by Titan Spine technology has been shown to induce a greater amount of osteogenic and angiogenic growth factors compared to conventional titanium PEEK cages [22]. This breakthrough demonstrates the potential that nanotech offers in improving the topographical interaction between implant and bone to increase osteogenesis.

Use of rhBMP-2 is commonly associated with side effects due to supraphysiologic dosing [23]. Nanotechnology efforts are underway to address these limitations. One particular strategy uses nanofiber structures known as peptide amphiphile (PA) molecules to mimic extracellular filaments and induce cellular regeneration. Studies found that the use of PA nanofibers in the form of a gel scaffold showed overall superior fusion rates while allowing for reduction of therapeutic doses of BMP-2 by up to 10-fold [24, 25]. Further efforts are investigating the reliability of this technique in promoting osteogenesis in vivo and ultimately its potential use as a growth-factor substitute in spinal fusion surgery [24].

## Orthopedic oncology

### Therapeutic applications - drug delivery

**Fig. 3:**
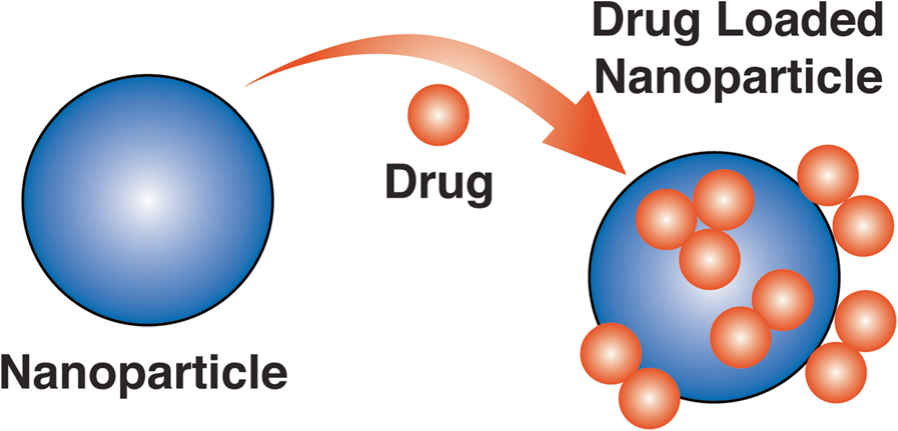
Diagram demonstrating the fundamental principle of drug delivery using nanoparticles. This method not only allows for more precise drug targeting, but also greater control of drug release in treating bone cancer, prosthetic joint infections, and osteomyelitis

Substantial progress has been made in the treatment of osteosarcoma and Ewing sarcoma in terms of prolonging survivorship, but major challenges still remain including cytotoxicity and decreased selectivity of chemotherapies, drug-resistance, and pharmacokinetic problems [26]. Nanotechnology may offer a solution to some of these issues through the use of unique carrier molecules that aid in drug delivery. The principle behind nanotechnology drug delivery as a treatment modality begins with the creation of a drug-loaded nanomolecule (Fig. 3). Next, this structure is attached to a specific ligand such as a monoclonal antibody, which is able to bind and enter the cancer cell. This allows for direct action of the chemotherapy on the desired target with reduced collateral toxicity to non-cancerous cells [27]. There are a wide variety of carrier materials including titanium, gold, calcium phosphate, and chitosan that are under investigation for use as nanoparticle drug carriers [28]. Lipid nanoparticle carriers offer an attractive method of treatment against osteosarcoma, as they have been shown to have excellent bioavailability and can be orally administered [29]. Conventional chemotherapeutic agents such as etoposide have been investigated using this technique in treating other types of cancer and have shown promising results against bone metastases [30, 31]. Silica nanocarriers enhanced with Zoledronate and loaded with doxorubicin exhibited significant cytotoxic activity against boney metastasis as well [32]. Most recently, Liu et al. developed a gambogic acid and retinoic acid loaded nanoparticle that induced a remarkably higher rate (28%) of apoptosis in osteosarcoma cells than conventional drug delivery [33]. Additionally, Zhou et al. demonstrated that the use of tailored nanocarriers loaded with cisplatin resulted in reduced renal accumulation and side effects compared to the free form while maintaining optimal anti-tumor activity against osteosarcoma cells [34]. Despite the encouraging results demonstrated by these novel drug carrier methods using nanotechnology, this research is very much still in its early stages.

### Therapeutic applications - anti-cancerous materials

It is common for patients who have undergone bone cancer resections to receive orthopedic implants. However, standard materials are not designed to inhibit the growth or recurrence of cancer. Therefore, efforts are underway to design implants that will encourage normal bone growth while preventing cancer growth. Selenium has been shown to exhibit these properties in the past, and nano-selenium implants have been demonstrated to inhibit the growth of malignant osteoblasts while promoting healthy bone function at the implant-tissue interface [35]. It was identified that the selenium nanomaterial, unlike untreated titanium implants, increased bone adhesion, calcium deposition, bone proliferation, and alkaline phosphatase activity. More recently, nanostructured magnesium alloy implants demonstrated anti-tumor properties after being enhanced through grain refinement. Human osteosarcoma cells were less viable and adhesive to this material [36].

## Diagnostic applications

The role of nanotechnology in cancer diagnosis is based on the binding of nanoparticle-ligand complexes to specific genetic mutations that allow for detailed imaging at the cellular level. The addition of a contrast agent to these complexes allows for visualization of the tumor cells that express the specific mutation [27]. This technique has been studied using the p15 gene, a tumor marker mutation commonly associated with lung metastasis in osteosarcoma [37]. Utilization of this practice may allow for early identification of the metastatic potential of a malignancy. Coupled with nanotechnology drug delivery, chemotherapy can be initiated before clinical symptoms appear to decrease patient morbidity. Additionally, the detection of nanomaterials using fluorescent probes may aid in the assessment of cancer response after therapy [38]. This method potentially offers higher accuracy in the calculation of the amount of tumor remaining than histologic analysis after tumor resection [39].

## Arthroplasty

### Implant material

**Fig. 4:**
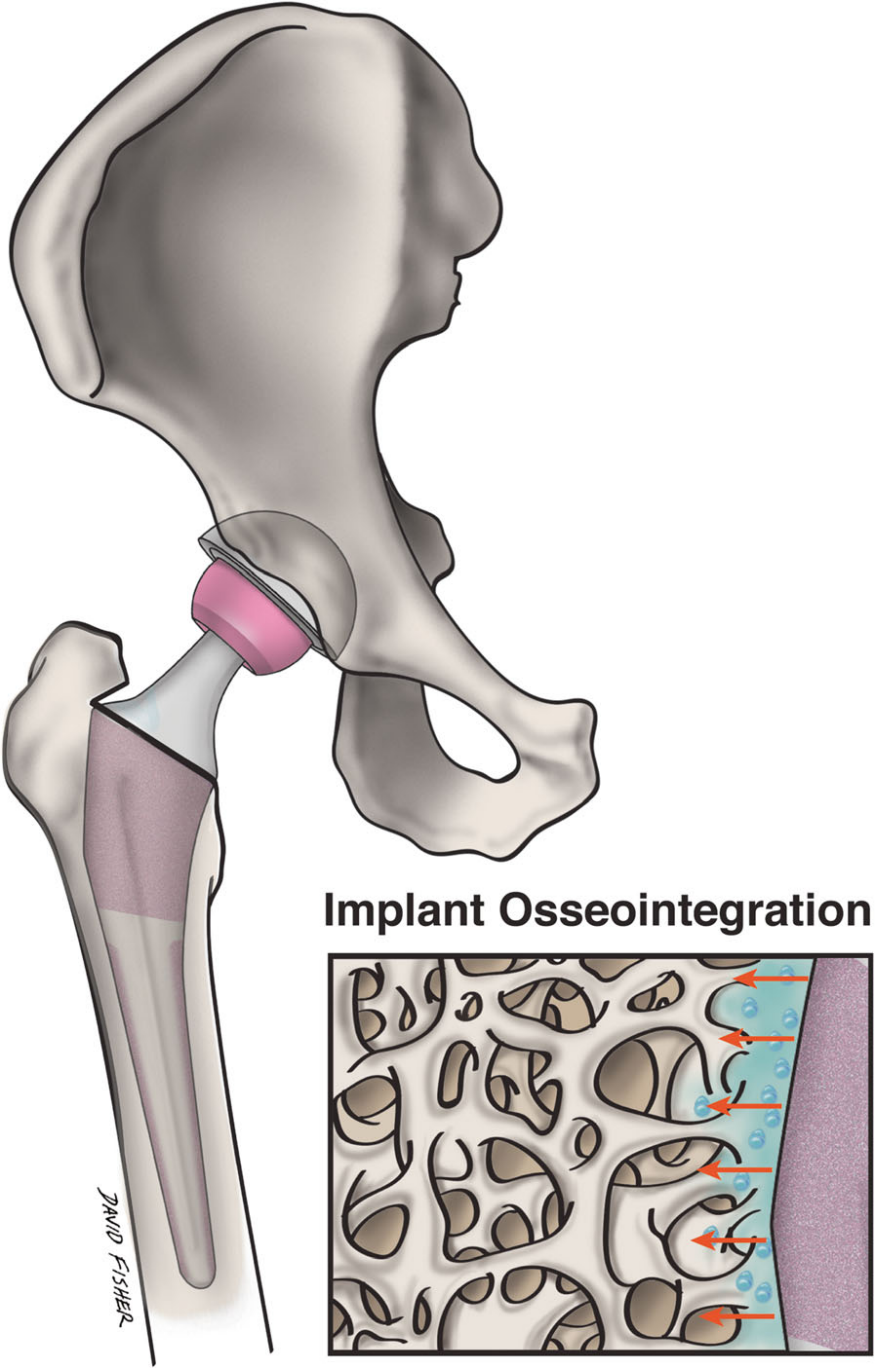
Nanostructured implants may better mimic the environment of native bone, and stimulate implant osseointegration and surrounding osteogenesis to a greater degree than conventional implants. This illustration shows a magnified nanoengineered implant surface and its topographical interaction with adjacent bone

Although primary joint replacement surgery is highly successful, its longevity remains limited. Nanotechnology in arthroplasty is focusing on the development of implantable materials that can function safely and effectively while extending the average lifespan of implants and preventing infection. Through the modification of specific surface characteristics on the implant, a more favorable interaction can be induced between the implant and native bone (Fig. 4). Nanotextured implant surfaces have augmented the function and growth of osteoblasts to increase implant osseointegration [5]. Specifically, the technique of severe plastic deformation (SPD), which breaks down the coarse grains of metals into the nanoscale range by exposing the metal to a complex high stress state, has demonstrated the ability to improve the biocompatibility and mechanical properties of titanium implants [40]. The use of ultra-high molecular weight polyethylene (UHMWPE) implants has been limited in the field of arthroplasty due to concern for potential fracture. However, due to its favorable biocompatibility properties and wear resistance, there has been increased interest in improving the mechanical strength of UHMWPE through nanotechnology. The addition of carbon nanotubes to this material to create a novel composite has demonstrated translational success and may eventually have utility as an acetabular lining or tibial component [41]. Altering an implant’s surface nanostructure has the potential to increase resistance to static and dynamic fatigue, improve functionality, and increase implant survivorship.

### Cements

Efforts to improve commonly used bone cements such as polymethyl methacrylate (PMMA) using nanotechnology are currently underway. Addition of antibiotics to bone cement is common practice, however it is well known that antibiotics often persist for only a short period of time ([42] Swearingen). Nanotechnology-based antibiotic carriers such as lipid nanoparticles [43], silica [44], and clay nanotubes [45] added to common cement material such as polymethyl methacrylate (PMMA) may enhance drug delivery and allow for timed release. Other types of non-antibiotic based nanotechnology cement additives such as chitosan, silver, and dendrimer are also under investigation for their anti-microbial properties [46]. Additionally, PMMA is well-known for eliciting an autoimmune response that can potentially lead to implant failure through fibrous encapsulation and inflammation [47]. Studies have found that the addition of nanostructured additives to PMMA demonstrated increased osseointegration and osteoblast activity [48, 49]. Ceramic particles such as zirconia and barium sulfate are often added to cements to allow for x-ray visualization, but these particles have a negative impact on the biocompatibility at the bone-implant interface [50]. Gilliani et al. showed that nanoscale modification of these particles added to bone cements increased cytocompatability and decreased mechanical failure [50]. Collectively, these results demonstrate the positive impact nanotechnology may have on improving the efficacy of bone cements.

## Sports medicine

### Chondrogenesis

**Fig. 5:**
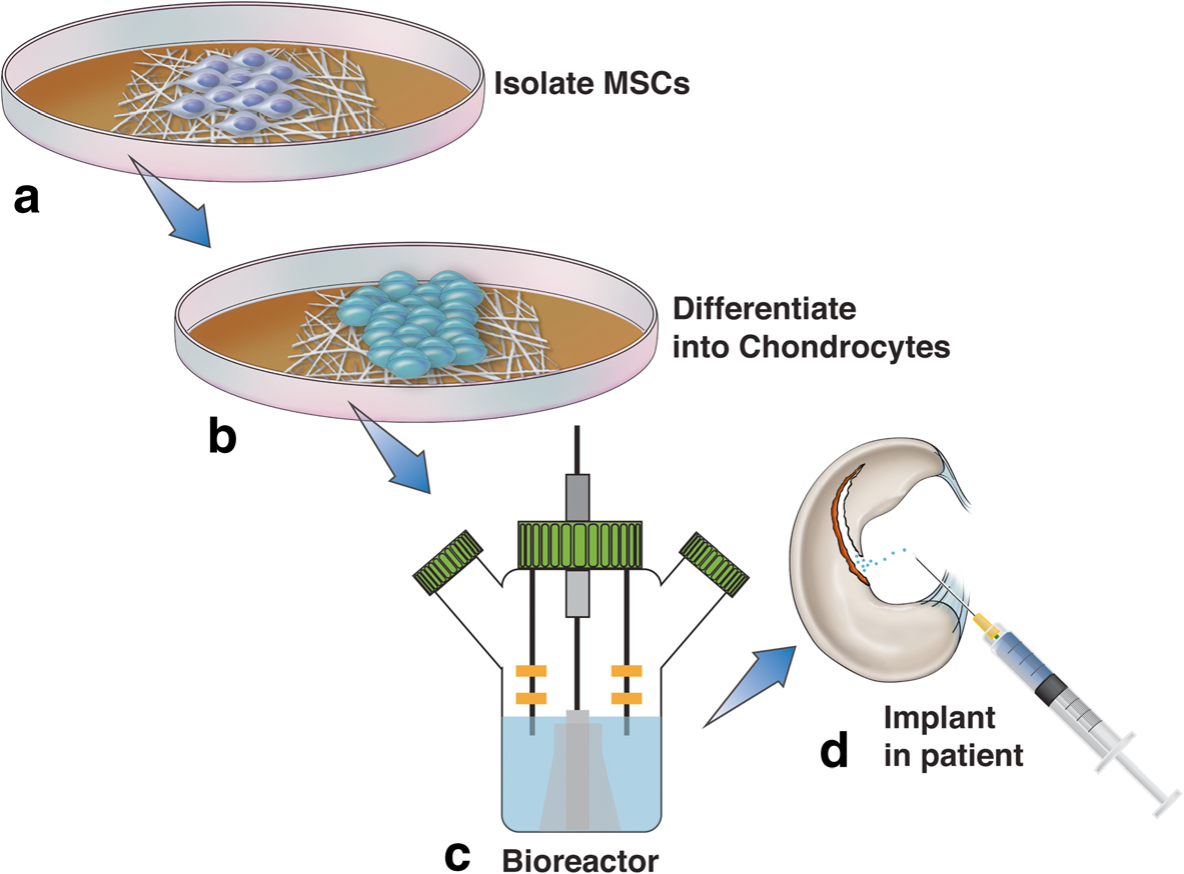
Regenerative techniques using human MSCs to treat osteochondral defects have had limited success, however nanotechnology may enhance the efficacy of these therapies. This diagram demonstrates the typical sequence of events for MSC treatment using nanotechnology. MSCs are first harvested from the patient and cultured in a growth medium (**a**). Once differentiated into chondrocytes (**b**), these cells are implanted onto the desired scaffold material, cultured in a bioreactor (**c**), and reimplanted into the patient (**d**)

Repair of cartilage defects is a topic that has been under extensive investigation in the field of regenerative medicine. Adult cartilage tissue lacks the proper repair response needed for complete regeneration, which if left untreated will undergo progressive degeneration to osteoarthritis. Preclinical efforts using nanotechnology to augment MSC therapy (Fig. 5) by developing a biomcompatible scaffold that enhances native cartilage repair have seen early success. [51–56] Yaylaci et al. designed a hyaluronic acid analogue using nanofibers to facilitate MSC differentiation towards the proper chondrogenic lineage without associated toxicities of natural scaffolds [53]. Liu et al. designed a nanofibrous scaffold composed of polycaprolactone and gelatin that enhanced articular cartilage repair and subchondral bone regeneration using pluripotent stem cells [54]. Recently, Mahboudi et al. demonstrated that the use of nanofiber-based polyethersulfone scaffold significantly enhanced chondrogenic differentiation of MSCs [51]. Aside from the aforementioned studies, a wide variety of other scaffolds including injectable hydrogels [57] and peptide-based materials [52] are under investigation for treatment of cartilage defects. One pilot study involving 28 patients with osteochondral defects showed 70% of defects were completely filled at two-year follow-up using an osteochondral nanoscaffold graft [56]. Other clinical trials have demonstrated mixed results at 3 years follow-up [58], however further studies are underway to investigate the efficacy and safety of these scaffolds. Although nanotechnology in cartilage regeneration has not yet achieved widespread clinical use, the utilization of nanomaterials as scaffolds for regenerative tissue engineering has been shown to favorably affect cell adhesion, proliferation, and phenotypic selection of chondrocytes [59].

### Tendon healing

Adhesion formation after tendon surgery remains a significant problem even with recent advances in surgical technique and post-operative care. Advances in nanotechnology and drug delivery may offer an appealing alternate to improve extrinsic and intrinsic tendon healing. Zhao et al. developed a strategy to allow for controlled release of mitomycin-C, a chemotherapy agent with the ability to decrease post-operative adhesions using hydrosol nanoparticles as drug carriers [60]. This allowed for reduction of tendon adhesion formation in vivo while maintaining comparable mechanical strength to naturally healed tendons. Tendon tissue engineering, particularly the through use of nanocomposite scaffolds, is another innovation that highlights the potential clinical value of nanotechnology in tendon healing. A plethora of different scaffolding materials are under investigation. Studies have shown that these scaffolds facilitate improved healing and mechanical stability and presentably fit the needs of regenerating tendons better than allografts [61–65]. Sharif-Aghdam et al. prepared a modified silk nanoscaffold that demonstrated excellent collagen content production and viability [66]. Huegel et al. showed that rat shoulders treated with autologous nanoscaffolds during supraspinatus repair surgery exhibited improved healing and mechanical stability [61]. Although tendon healing using nanotechnology has not yet reached the stage of clinical trials, research studies using tissue engineering techniques to simulate the bone-to-tendon interface are rampant [67–69].

## Musculoskeletal trauma

### Osteogenic properties of materials

**Fig. 6:**
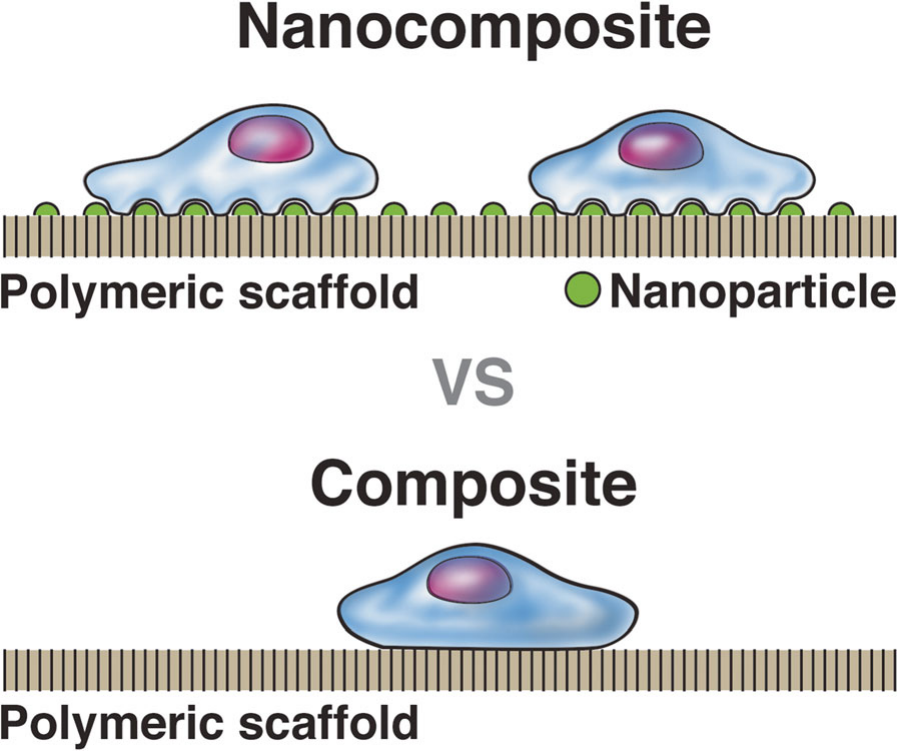
This illustration highlights the elemental layout of a nanocomposite scaffold compared to a conventional composite. The addition of nanoparticles to the general structure allows for more effective adhesion of surrounding osteoblasts

Similar to arthroplasty, nanotechnology research in the field of orthopedic trauma is focused on improving osseointegration of implants and promotion of healthy bone growth following fracture or non-union treatment. The key to the potential success of nanostructured implants in trauma is surface modifications that allow for better simulation of natural bone environment than conventional implants. The goal of many research efforts is to engineer a bioactive scaffold for bone regeneration that will allow for faster healing time and recovery of function (Fig. 6). Nanofiber scaffolds have been observed to improve cell migration and growth during bone healing and many studies have demonstrated the osteogenic capability of these nanomaterials [70–74]. Nanostructuring of an array of materials including polymers, ceramics, composites, and metals are under immense investigation but are not yet used clinically due to unanswered concerns regarding clinical safety. Nanotechnology may also have the ability to aid in the management of nonunion defects by providing an acceptable alternative to bone allografting. Nanoengineered synthetic grafts that mimic native bone structure have shown pre-clinical success in their ability to provide adequate mechanical stability and enhance osteoblast adhesion [75, 76]. Nanosilicates, an ultra-thin nanomaterial, may also be helpful in the healing of bony defects. They have demonstrated excellent bone stiffness, porosity, and mineralization when added to collagen-based hydrogels [77]. The capability of nanomaterials to improve osseointegration of orthopedic implants and enhance osteogenesis highlights the potential future utility of these materials in the clinical setting.

## Orthopedic infections

Infection remains a significant problem in the field of orthopedics and can lead to delayed healing, implant failure, and repeat surgery. Bacterial biofilms are often the source of infection and are definitively treated with implant removal. Therefore, recent efforts have focused on development of novel anti-biofilm implants equipped with nanoparticles. For example, titanium femoral stems incorporated with a novel vancomycin drug delivery system demonstrated sustained release for as long as 100 h [78]. Additionally, Besheli et al. showed that silk fibroin nanoparticles are effective in treating severe osteomyelitis in a controlled animal study [79].

Nanophase silver has become of significant interest in orthopedics over the past decade and is used clinically in wound care. Anti-microbial nanophase silver dressings have proven to be more effective at infection prevention and healing than conventional dressings [80]. Kose et al. developed a silver nanopowder coating that led to a decrease in bacterial colonization on coated titanium implants compared with uncoated [81]. Novel efforts investigating IL-12 as nanocoatings have shown promise in preventing open fracture-related infections, and may modulate immune responses to prevent infection [82]. Most recently, researchers developed a titanium pedicle screw coated with silver nanoparticles which has inhibited biofilm formation on the implanted screws in rabbits [83]. Overall, nanotechnology infection control efforts have demonstrated substantial promise to prevent acute post-operative infections in trauma and spinal implants in addition to joint replacements.

## Potential concerns

Though early translational research efforts have demonstrated the incredible potential of nanomedicine, major barriers exist to its widespread implementation into orthopedic clinical practice. First, the long-term effect of nanomaterials on human health is poorly understood. Early research has shown that nanomaterials may be associated with brain and lung cytotoxicity, systemic inflammation, and oxidative stress [84]. However, other studies suggest that the products of nanomaterial metabolism may actually benefit bone and lung tissue health at the cellular level [85]. With such uncertainty comes the intense regulatory processes of clinical trials set forth by the United States Food and Drug Administration (FDA). In addition to the rigorous process of approval for human use, the monetary cost of clinical trials can amass hundreds of millions of dollars. The combination of these two obstacles may cause many medical device companies to be reluctant to invest millions of dollars in capital when adequate implants already exist on the market [86]. In total, only 3% of nanotechnology research funding since 2008 has gone towards investigating its health effects [3]. Taking these issues into consideration, extensive research will be needed to investigate potential toxicities of nanomaterials before they can become widely used clinically.

Another challenge is the mass production of nanomaterials. Some experts argue that the high volume manufacturing of materials less than three nanometers is not consistently reproducible due to the complex structural properties. Kelly et al. demonstrated that when these materials are mass-produced on such a small scale, there can be variation in the size of certain components as well as variation in the physical properties [87]. Hence, the low-cost, high-volume model of manufacturing may not be accomplishable with certain nanomaterials without sacrificing some degree of reproducibility.

## Conclusion

Although still in its infancy, nanotechnology has the potential to revolutionize diagnostics, treatment, and research in orthopedics. The success of nanotech in commercial and service industries supports the expectation that the field will eventually play a significant role in clinical practice. Nanotechnology has the capability to inexpensively replace many conventional therapies and provide a multitude of novel applications. Nanotechnology offers more precise treatment modalities that may lead to more effective and longer lasting implants, decreased infection rates, and improved bone and tendon healing. Through immense basic science research efforts, the theoretical benefits of nanomedicine are beginning to be realized, specifically within the field of orthopedics. However, further investigations are needed to fully understand the safety and potential of this exciting technology.
